# Perceptions and plans for prevention of Ebola: results from a national survey

**DOI:** 10.1186/s12889-015-2441-7

**Published:** 2015-11-16

**Authors:** Bridget Kelly, Linda Squiers, Carla Bann, Alexander Stine, Heather Hansen, Molly Lynch

**Affiliations:** RTI International, 701 13th Street NW. Ste. 750, Washington, DC 20005 USA; RTI International, 6110 Executive Boulevard, Ste. 902, Rockville, MD 20852 USA; RTI International, 3040 East Cornwallis Road, Research Triangle Park, NC 27709 USA

## Abstract

**Background:**

Literature suggests that Americans may have higher levels of perceived threat to Ebola than are warranted.

**Methods:**

We surveyed 1018 U.S. adults from a nationally representative Internet panel about their knowledge, perceived threat, and behavioral intentions during the 2014 Ebola outbreak.

**Results:**

Eighty-six percent of respondents knew that Ebola could be transmitted through blood and bodily fluids. However, a large percentage had some inaccurate knowledge and 19 % believed Ebola would spread to the U.S. Respondents favored mandatory quarantine (63 %) and travel bans (55 %). Confidence in the ability of the media and government to accurately report on or prevent a U.S. epidemic was low. Fifty-two percent intended to engage in behaviors such as avoiding public transportation.

**Discussion:**

Despite low perceived susceptibility, half intended to engage in behaviors to prevent transmission and large numbers favored policies not currently recommended by health officials. The extreme nature of Ebola virus likely motivated people to engage in behaviors and favor policies that were not necessary given the low risk of transmission in the U.S.

**Conclusions:**

Health officials should ensure the public has accurate information about Ebola and bolster confidence in the government’s ability to control infectious diseases in case of a future outbreak in the U.S.

**Electronic supplementary material:**

The online version of this article (doi:10.1186/s12889-015-2441-7) contains supplementary material, which is available to authorized users.

## Background

In the fall of 2014, the Ebola outbreak in West Africa was ongoing, with cases stabilizing in Guinea and beginning to decline in Liberia, but “gathering pace” in Sierra Leone [1]. At that time, West Africa had confirmed more than 18,000 cases and 6800 deaths. U.S. health officials had treated one patient diagnosed in the U.S., two healthcare workers infected in the U.S., and several U.S. citizens infected in West Africa and brought to the U.S. for treatment. The news media covered the epidemic heavily; one report indicated that between October 3 and November 5, the major news networks aired 1000 Ebola segments [2].

The U.S. public often turns to media, and particularly television, in an emergency. Depending on the content and framing of news stories, media coverage can have a significant impact on those who are exposed to it. Several theories of news media effects provide insight about how such effects occur. According to agenda setting theory, the media tells us *what* to think about by varying the prominence of certain issues in the news. Second-level agenda setting suggests the tone and perception used to cover an issue can also impact *how* we think about that particular issue [3–5]. The media may also use different frames to present issues in ways that will be most likely to resonate with their audience [6]. For example, a disease outbreak could be framed in terms of its economic impact, rather than its immediate health consequences. Social amplification of risk theory suggests that hazards or risks interact with psychological, social, institutional, and cultural processes in ways that may amplify or attenuate public responses to the risk [7]. Signals about risk are processed by individual and social amplification stations, including the news media and interpersonal networks. Attributes of information that may influence the social amplification include volume and the extent of dramatization [7], both particularly relevant in the case of Ebola news coverage.

In some cases, when content is complete and accurate, it can reduce levels of fear and anxiety [8]. However, some literature provides empirical evidence for social amplification, showing that overly frequent news coverage can lead to distorted perceptions of susceptibility and severity [9], essential constructs of the Health Belief Model [10]. In a 2008 experiment at McMaster University in Ontario, researchers asked undergraduates and medical students their impressions of ten infectious diseases. Five of the diseases had received more media coverage than the other five. The “high-media frequency” diseases were rated as more serious than the more obscure diseases. Both groups overestimated the chances they would get one of the frequently reported diseases [11].

In extreme cases, media coverage can increase fear to the level of panic. For example, in a 1994 outbreak of plague in India, the announcement of the disease provoked many people to flee the state of Surat, carrying the disease to other parts of the country [12].

Although news coverage may increase perceived susceptibility and severity, its effects on knowledge are more complex. Several studies have highlighted the high prevalence of misconceptions or low levels of knowledge despite seemingly ubiquitous news coverage [11]. Following the H1N1 pandemic, a survey of Arizona residents found 34 % were unaware the terms “swine flu” and “H1N1” referred to the same virus. Results of a systematic review of community response studies during that same period found awareness of the pandemic was high, although knowledge levels were only moderate [13].

Even less is known about preparedness behaviors during outbreaks. Literature on previous pandemics shows behavioral intentions for recommended behaviors like hand washing, can be high [14]. Many of these studies have been limited to specific geographic areas.

It is unclear what effect news coverage of Ebola in 2014 might have had on the American public. Although a number of polls conducted by news agencies have investigated the perceived threat, there remains a paucity of peer-reviewed literature on knowledge, perceptions, and behaviors related to Ebola in the U.S. The purpose of the survey described here was to determine what Americans knew about Ebola, how they perceived the threat, and what steps they had taken to protect themselves and their family members during the winter 2014 holiday season. Based on the literature and relevant theory, we hypothesized that Americans may have had higher levels of perceived threat than were warranted by actual risk. We measured attitudes and intentions as described in the Theory of Reasoned Action [15]. In addition, we assessed perceptions and beliefs about possible Ebola-related policies, such as mandatory quarantine and travel bans.

## Methods

### Data source

We recruited participants through an Internet panel maintained by GfK Custom Research, LLC. The GfK KnowledgePanel® consists of 50,000 adult panel members recruited by address-based sampling (ABS). The GfK KnowledgePanel® is based on probability sampling covering both online and offline populations in the U.S. GfK presents households with access to the Internet and a netbook computer, if needed. The resulting sample includes representation from listed and unlisted telephone numbers, telephone and non-telephone households, and cell phone-only households, as well as households with and without Internet access.

### Sample selection

Eligible participants were U.S. residents age 18 and older. A random sample of 3222 panel members was drawn from GfK’s KnowledgePanel®. A total of 1018 participants completed the survey, yielding a final stage completion rate of 33 %. The panel recruitment rate for this study was 13.8 % and the profile rate was 64.1 %, for a cumulative response rate of 2.8 %. It is important to note that response rates for online panels tend to be lower than for other modes, due to the need to multiply recruitment rate, profile rate, cooperation rate and retention rate for a cumulative response rate [16]. We calculated response rates based on standard formulas for online panel response rates [16].

### Data collection

The survey was completed as part of a larger survey with multiple topics and was fielded December 5–7, 2014. All procedures were approved by RTI International’s Institutional Review Board. GfK obtains online consent from all panelists at the time they are recruited to the panel and again before each individual survey.

### Measures

Measures are briefly described in this section. Additional file 1: Table S1 provides the full list of measures.

### Perceived susceptibility and perceived severity

Three measures assessed perceived susceptibility and severity. We asked how likely it was that the Ebola outbreak in West Africa would spread to the U.S. and how likely it was that the respondent or their community would be affected by Ebola. The measure of severity asked if someone in the respondent’s community were to contract Ebola, how likely they would be to die from the disease.

### Perceived threat

We asked respondents to rate the level of perceived threat for each item on a list of issues, including heart disease, the seasonal flu, a pandemic flu (bird flu, swine flu), and Ebola.

### Knowledge

Knowledge was measured with three items capturing how Ebola is spread and how long it could it take for someone to get sick after being exposed.

### Behavioral intentions

We measured behavioral intentions with a list of questions about social distancing and other protective measures.

### Attitudes and confidence

We also measured attitudes toward a number of Ebola-related policies such as mandatory quarantine and travel bans. We assessed confidence in media, government, and healthcare systems.

Other variables included respondent gender, age, race/ethnicity, education level, income level, U.S. Census region, and parental status. These items were collected as part of a profile completed by panelists upon joining the GfK KnowledgePanel®. These data are updated annually.

### Analysis

We calculated the percentage of respondents endorsing each attitude and confidence item by demographic characteristics. Demographic differences in responses were tested using logistic regression models to compute odds ratios adjusted for gender, age, education, race/ethnicity, income, presence of children in the home, and region. We conducted paired t-tests to compare differences in perceived threat and knowledge between Ebola and other issues. The survey data were weighted to represent the U.S. population based on the most recent Census reports. The survey weights were developed using an iterative proportional fitting procedure, utilizing the following demographic characteristics: gender, age, income, race/ethnicity, region, metropolitan statistical area (MSA), and Internet access. The survey weights were then incorporated into our statistical analyses using the survey procedures in SAS version 9.3.

## Results

### Demographics

Table 1 presents demographic characteristics of the sample.

**Table 1:** Demographics of the sample

	N	Unweighted %	Weighted %
Gender			
Male	501	49.2 %	48.7 %
Female	517	50.8 %	51.3 %
Age			
18–39	380	37.3 %	36.8 %
40–59	400	39.3 %	35.2 %
60+	238	23.4 %	28.0 %
Education			
HS or less	387	38.0 %	42.1 %
Some college	292	28.7 %	29.0 %
College or more	339	33.3 %	28.9 %
Race			
Black	78	7.7 %	11.6 %
White	760	74.7 %	65.9 %
Hispanic	109	10.7 %	15.0 %
Other	71	7.0 %	7.5 %
Income			
< $30,000	242	23.8 %	23.8 %
30,000–74,999	365	35.9 %	35.7 %
$75,000+	411	40.4 %	40.5 %
Children in home			
Yes	293	28.8 %	29.2 %
No	725	71.2 %	70.8 %
Region			
Northeast	186	18.3 %	18.1 %
Midwest	269	26.4 %	21.3 %
South	336	33.0 %	37.2 %
West	227	22.3 %	23.4 %
Total	1018	100.0 %	100.0 %

### Perceived susceptibility and severity

**Fig. 1:**
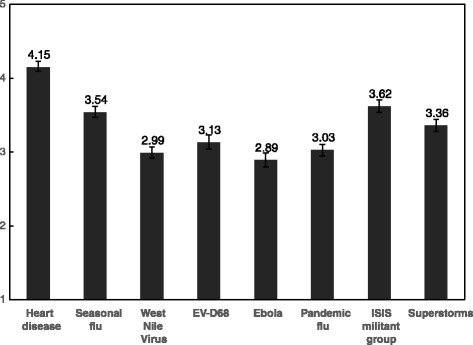
Perceived threat of eight issues in the news Perceived threat was measured on a five point scale (1 = no threat at all; 5 = a very serious threat). Note: T-tests for difference in means on perceived threat show differences between all issues vs. Ebola are statistically significant at *p* < .001 except for WNV, different at *p* < .01

Only 19 % of respondents believed it was likely or extremely likely that Ebola would spread to the U.S. (52 % believed it was unlikely; 29 % were neutral). Fig. 1 depicts the perceived threat of Ebola as compared to other issues in the news. Ebola was reported as less of a threat than heart disease or seasonal flu, but about the same level of threat as West Nile Virus (WNV). When asked about their own community, 77 % said it was unlikely their community would be affected by Ebola in the next few months, and 86 % thought it was unlikely they or their family would be affected. Asked how likely someone in their community would be to die from Ebola if they contracted it, 22 % said it was likely (42 % thought it was unlikely; 36 % were neutral).

### Knowledge

A number of participants had some inaccurate knowledge. Table 2 presents responses to the specific knowledge questions. When we created an index for knowledge (totaling all possible correct answers) the correlation of that index with confidence in one’s own ability to understand how Ebola is transmitted or how to protect themselves or their families was very low (*r* = −0.07).

**Table 2:** Responses to knowledge questions

Knowledge question	Answer option	N	%
To the best of your knowledge, which of the following are ways that Ebola can spread? (Check all that apply.)	Contact with bodily fluids of a person who has been exposed to Ebola but does not yet have symptoms;	614	60 %
	Contact with blood and bodily fluids of a person who is sick with Ebola;^a^	896	86 %
	Breathing the same air as a person who is sick with Ebola	217	22 %
	Touching public door handles, shopping cart handles, or public toilet seats	178	18 %
	Touching the body of someone who has died from Ebola.^a^	418	41 %
To the best of your knowledge, how long could it take for someone to get sick after being exposed to Ebola? (select only one response).	1–2 days (up to 2 days)	118	13 %
	Up to 21 days (up to 3 weeks)^a^	688	67 %
	Up to 28 days (up to 4 weeks)	156	16 %
	More than 28 days (more than 4 weeks)	37	4 %
Which of the following statements do you believe is true? (Select all that apply):	Ebola can only be spread once a person has symptoms^a^	651	64 %
	Mosquitoes spread Ebola	121	12 %
	There is a new vaccine available for widespread use that can prevent someone from getting Ebola	121	12 %
	You should avoid food and drinks imported from West Africa to prevent contracting Ebola	174	17 %
	You can get Ebola from your cat or dog	103	10 %

^a^Correct answers

### Intentions to engage in preparedness behaviors

Fifty-two percent of respondents reported engaging (or planning to engage) in one or more behaviors to prevent contracting Ebola. Forty percent reported intentions to engage in at least two of the behaviors. The most commonly reported were avoiding those who have traveled to West Africa (39 %) and making changes to hygiene practices such as hand washing (35 %), followed by avoiding public transportation during the holiday season (22 %), avoiding healthcare facilities (13 %), purchasing self-protective supplies (12 %), and keeping children home from school or avoiding public places (10 %).

### Attitudes toward quarantine, travel bans, and other policies

Sixty-four percent said anyone who has been exposed to an Ebola patient should be quarantined for 21 days whether they show symptoms or not (11 % disagreed; 25 % were neutral). Fifty-six percent said the U.S. should ban travel from affected countries in West Africa (18 % disagreed; 26 % were neutral). Twenty-seven percent reported the media has exaggerated the seriousness of Ebola (38 % disagreed; 35 % were neutral). Thirty-six percent felt healthcare workers who are infected with Ebola while treating patients in Africa should be brought to the U.S. for care (32 % disagreed; 33 % were neutral). There were some demographic differences. For example, men (OR = .75, 95 % CI = .57, .99), Blacks (OR = .55, 95 % CI = .33, .91) and those living in the Midwest (OR = .55, 95 % CI = .36, .83) and West (OR = .64, 95 % CI = .41, .98) were less likely to favor travel bans. Older participants (ages 40–59: OR = 1.60, 95 % CI = 1.15, 2.23; 60+ OR = 1.37, 95 % CI = .93, 2.03) and those with children in the home (OR = 1.42, 95 % CI = 1.03, 1.95) were more likely to favor travel bans (see Table 3).

**Table 3:** Demographic differences in attitudes towards various Ebola policies

	Favors quarantine		Favors travel ban		Supports bringing healthcare workers to U.S. for treatment	
Characteristic	N (%)	Adjusted OR	N (%)	Adjusted OR	N (%)	Adjusted OR
		(95 % CI)		(95 % CI)		(95 % CI)
Gender						
Male	309 (61)	0.81 (0.60, 1.08)	262 (52)	0.75 (0.57, 0.99)*	177 (34)	0.86 (0.65, 1.13)
Female	336 (66)	REF	301 (59)	REF	183 (37)	REF
Age						
18–39	220 (58)	REF	188 (50)	REF	125 (34)	REF
40–59	276 (69)	1.60 (1.15, 2.23)**	226 (56)	1.39 (1.01, 1.90)*	145 (36)	1.10 (0.79, 1.52)
60+	149 (64)	1.37 (0.93, 2.03)	149 (63)	1.91 (1.29, 2.82)**	90 (38)	1.24 (0.85, 1.82)
Education						
HS or less	250 (63)	REF	226 (58)	REF	123 (32)	REF
Some college	204 (72)	1.49 (1.03, 2.14)*	171 (60)	1.22 (0.86, 1.72)	91 (33)	1.10 (0.77, 1.58)
College or more	191 (57)	0.74 (0.51, 1.06)	166 (48)	0.73 (0.51, 1.05)	146 (44)	1.77 (1.23, 2.54)**
Race/ethnicity						
White	486 (65)	REF	429 (58)	REF	263 (35)	REF
Black	50 (66)	0.88 (0.51, 1.52)	37 (51)	0.55 (0.33, 0.91)*	31 (42)	1.50 (0.88, 2.56)
Hispanic	62 (56)	0.54 (0.42, 1.03)	57 (50)	0.64 (0.41, 1.01)	44 (38)	1.40 (0.89, 2.21)
Other	47 (67)	1.16 (0.61, 2.23)	40 (53)	0.80 (0.42, 1.52)	22 (30)	0.81 (0.43, 1.54)
Income						
< $30,000	150 (59)	REF	151 (60)	REF	74 (33)	REF
$30,000–$74,999	242 (68)	1.56 (1.05, 2.31)*	201 (57)	0.90 (0.61, 1.31)	120 (34)	1.05 (0.71, 1.56)
$75,000+	253 (63)	1.27 (0.85, 1.90)	211 (52)	0.75 (0.50, 1.11)	166 (39)	1.20 (0.80, 1.81)
Children in home						
Yes	192 (66)	1.20 (0.86, 1.69)	168 (58)	1.42 (1.03, 1.95)*	98 (34)	0.87 (0.62, 1.21)
No	453 (63)	REF	395 (55)	REF	262 (37)	REF
Region						
Northeast	100 (56)	REF	104 (61)	REF	64 (36)	REF
Midwest	174 (65)	1.32 (0.85, 2.03)	136 (49)	0.55 (0.36, 0.83)**	92 (35)	1.07 (0.69, 1.65)
South	228 (68)	1.69 (1.10, 2.58)*	202 (60)	0.90 (0.60, 1.35)	126 (37)	1.11 (0.73, 1.68)
West	143 (61)	1.15 (0.74, 1.79)	121 (52)	0.64 (0.41, 0.98)*	78 (34)	1.00 (0.64, 1.58)
Total	645 (64)	--	563 (56)	--	360 (36)	--

* *p* < 0.05, ** *p* < 0.01, *** *p* < 0.001; *REF* reference category; odds ratios are adjusted for gender, age, education, race/ethnicity, income, children in home, and region. Total ns in the table represent the number who answered four or five on the 5-point Likert scale (corresponding toz *N* = 1018

### Confidence in government, health officials and media

We asked respondents to rate their confidence in the ability of the government, public health officials, and the media to perform specific roles related to communicating about and managing the Ebola epidemic in the U.S. 28 % reported being confident or very confident in the ability of the U.S. government to prevent the spread of Ebola to the U.S. 32 % were confident in their local hospital’s ability to treat the illness, and 31 % were confident their local hospital could prevent the spread to healthcare workers. Just 18 % were confident in the media’s ability to accurately report on the outbreak. Thirty-one percent said they were confident that public health officials were providing the U.S. public with all of the information they need to know about Ebola; 33 % were confident that the U.S. has provided the appropriate level of support to countries with Ebola outbreaks. Table 4 presents the overall means and the means among specific subgroups. Compared to women, men were less confident in their local hospital’s ability to prevent healthcare workers from contracting Ebola (OR = .73, 95 % CI = .54, .98) and in the media’s ability to accurately report on the outbreak (OR = .55, 95 % CI = .38, .79). Those in the South also had less confidence in the media’s ability to accurately report on the outbreak (OR = .59, 95 % CI = .36, .98).

**Table 4:** Demographic differences in Ebola perceptions and behavioral intentions

	Confident in the U.S. government’s ability to prevent spread of Ebola to U.S.		Confident in the media’s ability to accurately report on an Ebola outbreak		Confident that public health officials are providing U.S. public with all of the info they need about Ebola		Confident in local hospital’s ability to treat an infected patient		Confident in local hospital’s ability to prevent healthcare workers from catching Ebola		Confident the U.S. has provided the right level of support to countries with Ebola outbreaks	
Characteristic	N (%)	Adjusted OR	N (%)	Adjusted OR	N (%)	Adjusted OR	N (%)	Adjusted OR	N (%)	Adjusted OR	N (%)	Adjusted OR
		(95 % CI)		(95 % CI)		(95 % CI)		(95 % CI)		(95 % CI)		(95 % CI)
Gender												
Male	142 (29)	1.04 (0.77, 1.41)	68 (13)	0.55 (0.38, 0.79)**	145 (29)	0.78 (0.58, 1.05)	148 (31)	0.89 (0.67, 1.19)	139 (28)	0.73 (0.54, 0.98)*	159 (32)	0.96 (0.72, 1.29)
Female	139 (28)	REF	106 (22)	REF	167 (34)	REF	166 (33)	REF	169 (33)	REF	165 (33)	REF
Age												
18–39	105 (28)	REF	60 (17)	REF	110 (29)	REF	123 (32)	REF	111 (28)	REF	114 (30)	REF
40–59	117 (32)	1.14 (0.81, 1.61)	69 (18)	1.11 (0.72, 1.69)	122 (32)	1.18 (0.83, 1.67)	120 (32)	0.92 (0.65, 1.29)	127 (33)	1.22 (0.87, 1.72)	127 (34)	1.20 (0.86, 1.67)
60+	59 (24)	0.84 (0.55, 1.28)	45 (18)	1.12 (0.68, 1.85)	80 (34)	1.32 (0.88, 1.98)	71 (32)	1.05 (0.70, 1.57)	70 (30)	1.11 (0.74, 1.68)	83 (34)	1.20 (0.81, 1.78)
Education												
HS or less	102 (27)	REF	82 (10)	REF	108 (28)	REF	106 (28)	REF	98 (25)	REF	127 (32)	REF
Some college	75 (28)	0.99 (0.67, 1.46)	51 (19)	0.96 (0.61, 1.49)	79 (29)	1.12 (0.76, 1.64)	91 (33)	1.26 (0.87, 1.83)	90 (32)	1.43 (0.98, 2.08)	86 (32)	0.98 (0.69, 1.40)
College or more	104 (32)	1.15 (0.78, 1.70)	41 (13)	0.62 (0.38, 1.01)	125 (38)	1.73 (1.17, 2.56)**	117 (36)	1.31 (0.89, 1.90)	120 (37)	1.59 (1.08, 2.33)*	111 (35)	1.13 (0.78, 1.64)
Race/ethnicity												
White	202 (27)	REF	116 (16)	REF	228 (31)	REF	225 (30)	REF	231 (32)	REF	240 (33)	REF
Black	24 (31)	1.23 (0.72, 2.11)	15 (19)	1.00 (0.53, 1.88)	25 (32)	1.05 (0.61, 1.83)	28 (38)	1.71 (1.00, 2.93)*	24 (30)	0.95 (0.56, 1.63)	23 (32)	0.97 (0.56, 1.70)
Hispanic	30 (27)	1.05 (0.65, 1.72)	24 (22)	1.29 (0.75, 2.23)	39 (35)	1.45 (0.91, 2.32)	36 (33)	1.30 (0.81, 2.08)	30 (26)	0.86 (0.53, 1.39)	35 (31)	1.02 (0.63, 1.63)
Other	25 (37)	1.61 (0.84, 3.12)	19 (25)	1.50 (0.75, 2.98)	20 (29)	0.99 (0.51, 1.93)	25 (36)	1.33 (0.70, 2.55)	23 (30)	0.91 (0.47, 1.75)	26 (39)	1.34 (0.72, 2.49)
Income												
< $30,000	63 (26)	REF	57 (24)	REF	75 (32)	REF	64 (25)	REF	64 (27)	REF	77 (33)	REF
$30,000–$74,999	90 (26)	0.97 (0.64, 1.46)	52 (15)	0.62 (0.39, 0.98)*	99 (28)	0.81 (0.55, 1.21)	105 (30)	1.31 (0.86, 1.98)	96 (27)	0.96 (0.64, 1.44)	111 (32)	0.98 (0.66, 1.45)
$75,000+	128 (32)	1.23 (0.80, 1.90)	65 (16)	0.76 (0.47, 1.24)	138 (34)	0.95 (0.63, 1.45)	145 (37)	1.79 (1.16, 2.77)**	148 (36)	1.32 (0.87, 2.01)	136 (34)	0.99 (0.65, 1.49)
Children in home												
Yes	79 (29)	0.96 (0.68, 1.37)	50 (19)	1.08 (0.71, 1.64)	82 (29)	0.88 (0.62, 1.25)	89 (31)	0.88 (0.63, 1.25)	88 (30)	0.96 (0.68, 1.35)	86 (31)	0.92 (0.66, 1.30)
No	202 (28)	REF	124 (17)	REF	230 (32)	REF	225 (32)	REF	220 (31)	REF	238 (34)	REF
Region												
Northeast	52 (29)	REF	35 (21)	REF	64 (36)	REF	51 (29)	REF	61 (34)	REF	61 (34)	REF
Midwest	75 (29)	1.05 (0.67, 1.66)	44 (17)	0.71 (0.42, 1.20)	80 (30)	0.82 (0.53, 1.28)	83 (33)	1.31 (0.82, 2.10)	79 (29)	0.82 (0.53, 1.28)	87 (34)	0.99 (0.64, 1.53)
South	91 (29)	1.07 (0.69, 1.65)	48 (15)	0.59 (0.36, 0.98)*	107 (33)	0.91 (0.60, 1.39)	105 (32)	1.24 (0.79, 1.92)	103 (31)	0.94 (0.61, 1.43)	101 (32)	0.92 (0.60, 1.39)
West	63 (27)	0.94 (0.58, 1.51)	47 (21)	0.92 (0.54, 1.59)	61 (27)	0.68 (0.43, 1.08)	75 (33)	1.27 (0.79, 2.04)	65 (29)	0.82 (0.52, 1.30)	75 (33)	0.94 (0.60, 1.47)
Total	281 (28)	--	174 (18)	--	312 (31)	--	314 (32)	--	308 (31)	--	324 (33)	--

* *p* < 0.05, ** *p* < 0.01, *** *p* < 0.001; *REF* reference category; odds ratios are adjusted for gender, age, education, race/ethnicity, income, children in home, and region. Total ns in the table represent the number who answered four or five on the 5-point Likert scale (corresponding to confident or very confident). The total *N* = 1018

## Discussion

This study examined knowledge of, perceived risk of, attitudes toward, and behavioral intentions related to Ebola soon after the media coverage of the Ebola epidemic in West Africa and the few cases in the United States. News coverage prior to the survey provides context that allows us to better interpret the survey findings. Towers et al. [17] found that news videos on two major news networks between mid-September and late October 2014 were highly effective at inciting public concern. Each Ebola-related news video inspired over 10,000 Internet searches and tweets. Basch et al. [18] found that only 4 % of news articles in the three most widely-circulated U.S. daily newspapers between September 17, 2014 and October 17, 2014 included content on precautions the public could take. Although information about the nature of the news coverage prior to our survey provides some indication of the type of information to which the public was exposed, because we used a cross-sectional study design, we cannot conclude that the findings from this survey are a direct result of this media coverage.

Only 19 % of survey respondents believed Ebola would spread to the U.S. This is significantly less than reported in surveys conducted earlier in the fall [19]. If social amplification of risk occurred through heavy media volume, the reduction in media coverage that followed the first week of November [2] could possibly have contributed to this decline in perceived risk. One report suggests the amount of coverage decreased from 1000 segments in the 4 weeks prior to the mid-term elections to only 50 in the 2 weeks after [2]. The reduction in risk might also be explained by the fact that there were no longer any Ebola cases in U.S. hospitals and transmission to others within the states had been very limited.

With regard to relative risk, participants rated Ebola as less of a threat than many of the other issues, with heart disease, seasonal flu, ISIS, and superstorms rating highest and Ebola rating more similarly to pandemic flu, West Nile Virus, and EV-D68 (though differences between these issues and Ebola were still statistically significant). T-tests for difference in means on perceived threat show differences between all issues and Ebola are statistically significant at *p* < .001 (except for West Nile Virus, different at *p* < .01). Twenty-seven percent reported having no knowledge of EV-D68 (compared to only 2 % who had not heard of Ebola). Of those who had heard of EV-D68, the level of self-reported knowledge was significantly lower than for Ebola. This gap is striking, because in 2014 EV-D68 affected significantly more people in the U.S. (1121 people and 12 confirmed deaths) [20], than Ebloa (seven cases and two deaths). Without minimizing the crisis in West Africa, this knowledge gap suggests that media coverage of these health threats may have been unbalanced.

Despite seemingly reasonable notions of perceived risk of Ebola, slightly more than half of those surveyed said they planned to take some form of action to avoid contracting the illness, including either avoiding public transportation, avoiding those who have traveled to affected areas, or changing hygiene practices. This contrast between perceived susceptibility and behavioral intentions contradicts traditional notions of health behavior theory, which suggest people are most likely to act when perceived susceptibility is high [21–23]. The extreme and graphic nature of an hemorrhagic illness like Ebola may induce fear that motivates the behavior. Literature has shown that fear can be a very compelling motivator of health behavior [23, 24]. Although these behaviors may not have been recommended, they could have positive consequences in some cases. For example, changing hygiene practices can include hand washing, which can also reduce transmission of other communicable diseases.

Only 22 % of respondents said that if someone in their community were to contract the illness they would be likely to die. This finding is not in line with known Ebola case fatality rates (about 50 % in the 2014 outbreak) [25], but may have been influenced by the high survival rate for healthcare workers treated in the U.S.

Despite the frequent news coverage [2], the findings regarding knowledge make it clear that many Americans still do not have a clear understanding of how to avoid contracting Ebola. The low correlation between confidence in ability to understand Ebola and actual knowledge is not surprising; this is consistent with other literature [26, 27]. These findings suggest public health officials and the media need to ensure that communication about an outbreak also educates the public about basic issues like transmission and prevention, which should be communicated clearly, in non-technical terms, and repeated often [28, 29]. Public health officials can also use formative research to help identify which channels are most likely to reach the target audience in large numbers.

Responses to the questions about government policies favored conservative choices. Most respondents were in favor of mandatory quarantine for those who have been exposed and in favor of travel bans, which are not currently recommended. Participants were largely split on whether infected American healthcare workers should be brought to the U.S. for care. Demographic differences varied depending on the policy. For example, the same groups who favored quarantine were not most in favor of travel bans. It is unsurprising that those who are older or who have children in the home were more likely to favor travel bans. Older adults may be more conservative in general. Older adults and children could also be perceived as more susceptible to disease in general. Blacks were less in favor of travel bans from affected regions. Some research has found racial bias in ability to empathize with others’ pain [30]. Those in the Midwest and West were less in favor of travel bans than those in the Northeast. Those in the Northeast may have felt more vulnerable to infection because most points of entry from West Africa are located there. Patterns for those favoring quarantine are less clear. These differences should be explored in qualitative research.

Confidence in media, government, and local healthcare was low. This finding may be related to the fact that two nurses who had cared for a patient with Ebola at a Texas hospital contracted the disease just a few months prior to the survey. Because trust is one of the cornerstones of outbreak communication [31], and the media is the primary communication outlet for updating the public, public health officials and the media must develop concrete communication strategies to build Americans’ trust and confidence in order to be credible and prepared for future outbreaks. The Centers for Disease Control and Prevention have developed numerous materials on Crisis and Emergency Risk Communication (CERC), which include specific guidelines about communication during times of outbreak, such as acknowledging uncertainty, expressing empathy, and avoiding jargon. These can be valuable resources for building that trust [28]. Other strategies include being aware of the preexisting low levels of trust among the American public, being honest and open, providing enough information to make informed personal decisions, and not using euphemisms because they imply a lack of honesty [28, 29].

SteelFisher, Blendon, and Lasala-Blanco [27] recommend that for future outbreaks, public health officials should establish relationships with independent health professional associations that are trusted by the public and work with them to shape policies and messages with the public [29]. Finally, the media in particular must make sure that news stories provide an accurate portrayal of the risk of an infection or disease and not use gratuitous video footage just to secure high ratings.

Some demographic groups did have more confidence in these institutions than others. Some of these differences were not intuitive. It is not obvious why men might have less confidence than women in the media’s ability to accurately report on an Ebola outbreak, though this is consistent with previous research showing women tend to rate the media as more credible [32]. Differences in confidence in hospitals were somewhat nuanced. The groups who had more confidence in a local hospital’s ability to treat an infected patient did not necessarily have more confidence in the hospital’s ability to prevent healthcare workers from catching Ebola and vice versa. Perhaps tailored communications could help to reach the populations with lowest levels of trust.

Future research should also explore the implications of intentions to avoid those who have traveled to West Africa or foods and beverages produced there. Previous research has found significant social stigma associated with Ebola [33]. These findings could have serious implications for African people living in or traveling to the United States.

### Limitations

As is typical for online surveys using nationally representative panels, the response rate for the survey is less than ideal. However, for comparison, other surveys using GfK’s KnowledgePanel®, such as those conducted by Pew Charitable Trust and the Federal Reserve, have also reported response rates in the single digits [34, 35]. As was mentioned previously, response rates for panel surveys do tend to be lower than for other modes, due to the multiplication of retention, cooperation, profile and completion rates [16]. In addition, weighting helps to alleviate some concerns regarding the sample.

The data are self-reported, and there is likely some social desirability bias, which was not measured. Analysis of travel volume data, point of purchase data (for self-protective gear), and information from other more objective sources can help to shed light on the true impact of the epidemic on specific behaviors.

It is important to point out the variation that exists in the content of media information sources. Effects may have been different for audiences of different types of programming or different channels. It is possible some of the effects of age are due to the fact that younger people get more news online while older people watch more television news [36]. Future research should explore these differences further.

It is possible it was difficult for people to separate the seriousness of Ebola in West Africa from that in the U.S. when answering questions about whether the media had exaggerated its seriousness. Because these were not asked separately for the two regions, we cannot tease that out in this study.

## Conclusions

Findings from this study can be used to evaluate the effectiveness of the communication efforts by government agencies and the media to educate the public about Ebola and how it can be transmitted and prevented. Future outbreak communication efforts can avoid propagating misinformation and distrust by providing accurate, clear, and factual information through credible sources. Such efforts will help to prevent discrimination and ensure Americans know how to effectively protect themselves in the future.

## Additional file

Additional file 1:
**Measures.** Describes details of all measures reported on in the paper. (DOCX 17 kb)

## Abbreviations

ABS: Address-based sampling
EV-D68: Enterovirus D68
U.S.: United States
