# Supplementary material for: Perceptions and plans for prevention of Ebola: results from a national survey
Source: BMC Public Health. 2015 Nov 16;15:1136. doi: 10.1186/s12889-015-2441-7 (PMC4647489; doi:10.1186/s12889-015-2441-7)
Supplement: Additional file 1: — Measures. Describes details of all measures reported on in the paper. (DOCX 17 kb) [file 12889_2015_2441_MOESM1_ESM.docx]

# Supplemental Table 1. Measures

| **Construct** | **Measure** | **Response options** |
| --- | --- | --- |
| Perceived susceptibility | How likely is it that the recent Ebola outbreak in Africa will spread to the United States? | 1=not at all likely  2  3  4  5=extremely likely |
|  | How likely do you think it is that you or your community will be affected by Ebola in the next few months? (For example, healthcare facilities treat an Ebola patient, schools close, people who have been exposed to Ebola are quarantined; etc.)? | Respondents provided separate answers for “you or your family” and “your community”:  1=not at all likely  2  3  4  5=extremely likely |
| Perceived severity | If someone in your community contracted Ebola, how likely do you think they would be to die from the disease? | 1=not at all likely  2  3  4  5=extremely likely |
| Perceived threat | To the best of your knowledge, how serious a threat is each of the following for Americans?” (Select only one response for each):  heart disease  the seasonal flu  a pandemic flu (bird flu, swine flu)  Ebola  ISIS militant group in the Middle East  West Nile Virus  Superstorms (such as Hurricane Sandy)  Enterovirus D68 (EV-D68).  (Issues were presented in random order.) | 1 = not a threat at all  2  3  4  5 =a very serious threat  I don’t know/have not heard of this |
| Knowledge | To the best of your knowledge, which of the following are ways that Ebola can spread? (Check all that apply.) | Contact with bodily fluids of a person who has been exposed to Ebola but does not yet have symptoms;  Contact with blood and bodily fluids of a person who is sick with Ebola;*  Breathing the same air as a person who is sick with Ebola;  Touching public door handles, shopping cart handles, or public toilet seats; Touching the body of someone who has died from Ebola.*  *Correct answers |
|  | To the best of your knowledge, how long could it take for someone to get sick after being exposed to Ebola? (select only one response). | 1–2 days (up to 2 days)  Up to 21 days (up to 3 weeks)*  Up to 28 days (up to 4 weeks)  More than 28 days (more than 4 weeks)  *Correct answer |
|  | Which of the following statements do you believe is true? (Select all that apply): | Ebola can only be spread once a person has symptoms*  Mosquitoes spread Ebola  There is a new vaccine available for widespread use that can prevent someone from getting Ebola  You should avoid food and drinks imported from West Africa to prevent contracting Ebola  You can get Ebola from your cat or dog  *Correct answer |
| Behavioral intentions | Have you done or do you plan to do any of the following as a result of the Ebola outbreak? (Select only one response for each.)  Avoid public transportation during this holiday season (plane, train, bus)?; cancel travel to affected areas (West Africa)?; Keep children home from school or stay home from church, malls or other public places; purchase self-protective supplies (e.g., masks, gloves, protective clothing)? Stay away from people who have traveled to Africa; change or increase the frequency of hygiene practices (hand washing, use of anti-bacterial gels)?; avoid healthcare facilities (hospitals, doctor’s offices)?; other. | Yes  No |
| Attitudes toward Ebola-related policies | Please tell us how much you agree or disagree with the following statements: (Select only one response for each.)  (presented in random order)  Anyone who has been exposed to an Ebola patient should be quarantined (kept away from others) for 21 days, whether they show symptoms or not;  The U.S. should ban travel from affected countries in West Africa; Healthcare workers who are infected with Ebola while treating patients in Africa should be brought to the U.S. for care.  If I were exposed to Ebola, I would be willing to stay at home, away from other people for up to 21 days.  The media has exaggerated the seriousness of Ebola.  The U.S. should send troops to West Africa to help contain the illness. | 1=strongly disagree  2  3  4  5 = strongly agree |
| Confidence in media, government and healthcare | Please rate your confidence in the following: (Select only one response for each.):  (presented in random order)  The U.S. government’s ability to prevent the spread of Ebola to the U.S.;  The ability of health officials to contain (limit the spread of) an epidemic if one were to occur in the U.S.**;**  Your local hospital’s ability to treat an infected patient;  Your local hospital’s ability to prevent healthcare workers and others from contracting the disease from an infected patient;  The media’s ability to accurately report on an Ebola outbreak;  That public health officials are providing the U.S. public with all of the information they need to know about Ebola;  Your ability to understand how Ebola is transmitted and how to protect yourself and your family;  That the U.S. has provided the appropriate level of support to countries with Ebola outbreaks. | 1=not at all confident  2  3  4  5=very confident |
